# GeriMedRisk, a telemedicine geriatric pharmacology consultation service to address adverse drug events in long-term care: a stepped-wedge cluster randomized feasibility trial protocol (ISRCTN17219647)

**DOI:** 10.1186/s40814-018-0300-x

**Published:** 2018-06-20

**Authors:** Joanne Man-Wai Ho, Jennifer Tung, Janine Maitland, Derelie Mangin, Lehana Thabane, J. Michael Pavlin, Jeffrey Alfonsi, Anne Holbrook, Sharon Straus, Sophiya Benjamin

**Affiliations:** 1 Waterloo Regional Campus, McMaster University DeGroote School of Medicine, 10B Victoria St S, Kitchener, ON Canada; 2 Schlegel Research Institute for Aging, 250 Laurelwood Drive, Waterloo, ON Canada; 3 Grand River Hospital, 835 King St W, Kitchener, ON Canada; 4 St. Joseph’s Health Centre Guelph, 100 Westmount Ave, Guelph, ON Canada; 5 Department of Family Medicine, McMaster University, 6th floor, 100 Main St W, Hamilton, ON Canada; 6 Department of Health Research Methods, Evidence and Impact, McMaster University, H325, 50 Charlton Ave E, Hamilton, ON Canada; 7 Lazaridis School of Business and Economics, Wilfrid Laurier University, 64 University Ave W, Waterloo, ON Canada; 8 Ontario Telemedicine Network, 1100-105 Moatfield Drive, Toronto, ON Canada; 9 Division of Clinical Pharmacology and Toxicology, McMaster University, 1280 Main St W, Hamilton, ON Canada; 10 Li Ka Shing Knowledge Institute, St. Michael’s Hospital, 30 Bond St Toronto, Toronto, ON Canada; 11 Division of Geriatric Medicine, Department of Medicine, University of Toronto, 190 Elizabeth Street, R. Fraser Elliott Building, 3-805, Toronto, ON Canada

**Keywords:** Geriatrics, Appropriate prescribing, Telemedicine, Clinical trial, Feasibility, Cluster randomized controlled trial

## Abstract

**Background:**

Multimorbidity, polypharmacy, and older age predispose seniors to adverse drug events (ADE). Seniors with an ADE experience greater morbidity, mortality, and health care utilization compared to their younger counterparts. To mitigate and manage ADEs among this vulnerable population, we designed a geriatric pharmacology consultation service connecting clinicians with specialist physicians and pharmacists and will investigate the feasibility and acceptability of this complex intervention in the long-term care setting, prior to conducting a larger efficacy trial.

**Methods/Design:**

We will conduct a cluster randomized feasibility trial and qualitative analysis of GeriMedRisk among four long-term care homes in the Waterloo-Wellington region from May 1 to December 31, 2017. The primary outcome is the feasibility and acceptability of GeriMedRisk and the stepped-wedge cluster randomized controlled trial design. We hypothesize that GeriMedRisk is a feasible intervention and its potential to decrease falls and drug-related hospital visits can be evaluated with a stepped-wedge cluster randomized controlled trial design.

**Discussion:**

This mixed methods study will inform a larger efficacy trial of GeriMedRisk’s ability to decrease adverse drug events among seniors in the long-term care setting.

**Ethics and dissemination:**

The Hamilton Integrated Research Ethics Board granted the approval for this study protocol 2812. We plan to disseminate the results of this study in peer-reviewed journals and also to our partners and stakeholders.

**Trial registration:**

ISRCTN clinical trials registry, ISRCTN17219647 (March 27, 2017)

## Background

Every year, tens of thousands of seniors experience a poisoning or adverse drug event (ADE) [1, 2]. Multiple diseases, polypharmacy, and age predispose older adults to drug toxicity. Following a poisoning, seniors (aged ≥ 65 years) are four times more likely to die or to require hospitalization compared to their younger counterparts [3]. Drug toxicity is costly, with at least $13 million spent on ADE-related hospital visits among seniors and $419 million spent annually on potentially inappropriate medications in North America [1, 4]. Decreasing polypharmacy may prevent ADEs among seniors. Studies of deprescribing interventions among older adults found improved quality of life and decreased hospitalization and mortality [5]. Widespread application is limited, however, by inadequate systems of care integrating primary care, pharmacy, geriatric medicine, and clinical pharmacology [6, 7]. Pharmacist-led medication review services to assess medication appropriateness include the Medscheck and the Pharmaceutical Opinion Programs. The Ontario Pharmacy Research Network (OPEN), however, found that a minority of patients receiving a Medscheck were older and medically complex and that there was a discrepancy in the quality of the delivered service, and there was no evidence of improved patient outcomes [8]. Furthermore, existing deprescribing algorithms and apps may not apply to patients with multimorbidity or mental illness, a group vulnerable to ADEs [9]. The majority of expertise in geriatric pharmacology is concentrated in urban academic health sciences centers resulting in inequitable access for Canadians residing in rural and remote areas [10]. There is a need for a timely and cost-effective geriatric pharmacology consultation service for Ontario seniors irrespective of their location.

Telemedicine, or telehealth, is the use of communication technologies to deliver patient care and medical services remotely [11]. It enables the delivery of health care services to areas that are remote or that have insufficient clinicians and resources [11]. Telemedicine tools include telephone and computer- or mobile device-based videoconference or eConsult [11, 12]. Its use improves access to care, support from specialists to primary care, and potentially decreases health care costs [11, 13–15]. Telemedicine services have been able to increase access to specialist clinician services for toxicology through poison centers, stroke, psychiatry, dermatology, and hepatic disease [11, 16–19]. With the limited number of clinicians with specialization in geriatrics, telemedicine may be a potential solution to serve the increasing aging population [10, 14, 15].

GeriMedRisk is a novel interdisciplinary, technology-based geriatric pharmacology consultation service that aims to optimize a patient’s medications to improve cognition, mobility, function, and mental health by supporting their clinicians. Referring clinicians will be able to easily access GeriMedRisk nurses, pharmacists, and physicians specializing in geriatric medicine, clinical pharmacology, and geriatric psychiatry by telephone or through telemedicine. By supporting clinicians as they optimize their complex older patients’ medications, GeriMedRisk has the potential to decrease drug-related cognitive impairment, falls, and hospital visits among seniors from all clinical settings. Prior to conducting an efficacy trial of GeriMedRisk to decrease adverse drug events across 14 long-term care (LTC) homes in Southwestern Ontario, we will first test the feasibility of the service and proposed study design, a stepped-wedge cluster randomized controlled trial.

The aim of this study is to investigate the feasibility and acceptability of the GeriMedRisk and its evaluation with a stepped-wedge randomized controlled trial in the long-term care (LTC) setting. We also intend to identify indicators of a difference in falls and drug-related hospital visits among seniors residing in LTC with access to GeriMedRisk compared to those without. We hypothesize that GeriMedRisk is a feasible intervention, and its potential to decrease falls and drug-related hospital visits can be evaluated with a stepped-wedge cluster randomized controlled trial design.

## Methods

This protocol (version 4.0, March 27, 2017) was developed in concordance with the Standardized Protocol Item: Recommendations for Interventional Trials (SPIRIT) checklist and CONSORT extension [20] to pilot trials and is registered with the ISRCTN clinical trials registry (ISRCTN17219647). The trial sponsor is McMaster University located at 1280 Main St. W., Hamilton, Ontario, L8S 4L8, Canada.

### Setting

We will conduct this study across a convenience sample of four LTC facilities in the Waterloo-Wellington region, Ontario, Canada, with each site serving as a cluster. The Waterloo-Wellington region has a population exceeding 775,000 residents and encompasses the major urban centers of Waterloo, Kitchener, Cambridge, and Guelph. While 90% of the geography is rural, 90% of the population lives in urban areas. The population of Waterloo-Wellington has similar demographics as the rest of the province of Ontario [21]. At each site, residents are assigned to a primary care doctor or nurse practitioner. Each site is supported by a consultant pharmacist who performs medication reviews at full-time equivalents ranging between 0.25 and 0.3. The three Schlegel Village LTC homes have uniform physical environments and philosophies of care but individualized staffing and clinical programs. University Gates Schlegel Village is a 192-bed LTC home located in Waterloo, supported by primary care clinicians and regular on-site geriatric medicine and geriatric psychiatry consultants. Winston Park Schlegel Village located in Kitchener is a 95-bed facility supported by a primary care clinician and an on-site geriatrician consultant. Riverside Glen Schlegel Village is a 192-bed LTC home located in Guelph that does not have designated support from a geriatrician. LTC patients that require a geriatrician are referred through a centralized intake process through the Waterloo Wellington Specialized Geriatrics Services. St. Joseph’s Health Centre Guelph, a non-profit 244-bed facility, is supported by primary care physician and an on-site consultant geriatrician.

### Eligibility criteria

Inclusion criteria:

- Cluster: a convenience sample of LTC homes in Waterloo-Wellington region that expressed interest in participating in this feasibility study.
- Individual participants: all physicians, pharmacists, and nurse practitioners who provide patient care in a participating LTC site when they are allocated to the intervention.

Exclusion criteria:

- There are no exclusion criteria for LTC clinicians or residents.

Criteria for discontinuing the intervention:

- If the referring clinician’s patient status is an acute poisoning (defined as an ingestion of more than two times the prescribed dose), or clinically unstable, GeriMedRisk will abort the call and encourage the clinician to call either the Ontario Poison Centre and/or emergency services.

### Intervention

In addition to the usual Waterloo-Wellington regional geriatric services, clinicians caring for seniors with medication challenges will have access to GeriMedRisk. GeriMedRisk is an interdisciplinary, technology-based geriatric pharmacology consultation and review service. It aims to optimize a patient’s medications to improve cognition, mobility, function, and mental health by supporting their clinicians. Referring clinicians will be able to easily access GeriMedRisk nurses, pharmacists, and physicians specializing in geriatric medicine, clinical pharmacology, and geriatric psychiatry by telephone or through telemedicine during business hours. Input from the following informed GeriMedRisk’s development: long-term care residents and their caregivers; Waterloo-Wellington region health administrators; the Ontario Poison Centre, a provincial service that uses telemedicine to deliver toxicological expertise to the public and clinicians in Ontario, Canada; the Ontario Telemedicine Network; and Champlain BASE eConsult and a needs assessment of Waterloo-Wellington physicians, nurse practitioners, pharmacists about geriatric pharmacotherapy.

Participants have two options for methods for referral:

1. Telephone: this traditional mode of person-to-person consultation can be either real-time or delayed by a few hours if used as a paging service whereby the referring clinician leaves a call-back number.
2. eConsult: the referring clinician sends an eConsult with the patient’s necessary clinical information through a private and secure portal through the Ontario Telemedicine Network (OTN) at www.otnhub.ca to GeriMedRisk [12]. The GeriMedRisk team responds within five business days through eConsult with a request for additional information or recommendations. Additional support through the phone, OTN videoconference or in-person may be arranged if necessary.

The GeriMedRisk staff will collaborate with the referring clinician to perform a geriatric pharmacology consultation and medication review or answer a geriatric drug information question. The former process utilizes components from a comprehensive geriatric assessment to address the patient’s cognition, comorbidities, mobility, function, and mental health and incorporates an intrinsic medication appropriateness assessment [22, 23]. Videoconference telemedicine and in-person consultations with specialist physicians across Southwestern Ontario academic health centers (McMaster University, University of Toronto, University of Western Ontario) would be available if necessary. Password-protected consultation reports and user-friendly drug information and knowledge translation materials will be securely sent to the referring clinician through OTN eConsult. These educational materials are concise evidence-based documents relevant to the consult that serve to build geriatric pharmacotherapy capacity among referring clinicians. Their development is in accordance with rapid review methodology described by the National Collaborating Centre for Methods and Tools [24]. GeriMedRisk will perform at least one follow-up to the referring clinician within 2 weeks to assess the effectiveness of the recommendations and monitor adherence. During the duration of the study period, all LTC sites would continue to have access to their usual local and regional geriatric services. There will be no restrictions in concomitant care and interventions. The control period is defined as the 12-month period preceding the day the LTC’s clinicians acquired access GeriMedRisk.

### Study design

**Fig. 1:**
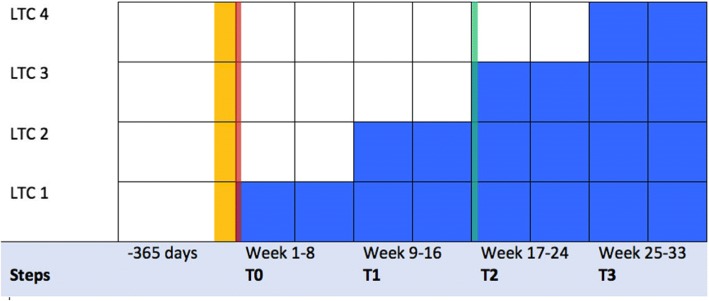
Schematic of study protocol. Each step from T0 to T3 will be approximately 8 weeks in length. Each cell starting at T0 represents a data collection point. Prior to T0, secondary outcomes will be collected retrospectively collected using the RAI-MDS 2.0. Yellow-shaded cells represent the recruitment period. During this time, LTCs will be invited to participate and will be provided trial information. Consent will be obtained from each LTC. Subsequently, investigator will provide in-person and online information sessions about the trial to potential participants (i.e., clinicians of the participating LTC site), and consent will also be obtained. Randomization (red line) will occur at the beginning of the first step, T0. The GeriMedRisk intervention (dark blue cells) will then be introduced to each LTC in random order. At the beginning of each step, a sealed envelope will be opened to reveal the next LTC to receive the intervention. An interim analysis (green line) will be performed at 16 weeks

We will conduct a mixed methods feasibility study using an exploratory framework. The quantitative component will be a stepped-wedge cluster randomized controlled trial. All study sites will have uniform access to their usual Waterloo Wellington Local Health Integration Network geriatric services. Using a computer-generated sequence and simple randomization techniques, we will randomize the order of when each site acquires access to GeriMedRisk. Every 8 weeks, a new site will gain access to the intervention until all four LTC sites have access to the intervention (Fig. 1). This time period allows for the orientation and potential adoption of GeriMedRisk and telemedicine technology prior to introducing the next site.

### Recruitment

At the commencement of GeriMedRisk access, there will be an initial informative site visit scheduled at a time to reach all the site’s LTC clinicians to introduce them to the study and GeriMedRisk including the various methods of accessing the intervention. Investigators will approach the participants (referring clinicians) in meetings facilitated by the long-term care facility at a time that is convenient for the participants. Flyers describing the intervention will be available for posting at the long-term care facility clinical staff areas once the LTC site (cluster) has access to the intervention. Although residents are not subjects of the study, we will notify them or their substitute decision-makers about the study upon the site’s introduction to the intervention until its conclusion and provide the option and instructions (LTC site-specific individual and their contact information) to opt out of the service.

### Outcomes

#### Primary outcome

Primary outcome measures of this trial are the feasibility and acceptability of the GeriMedRisk intervention, outcome measures, and study design. These include the monthly number and proportion of clinicians who use or decline GeriMedRisk per long-term care home, the number of patients or their substitute decision-makers who decline a GeriMedRisk consultation, and the number of consults, wait times for telephone (ring, queue and call time, or the number of dropped calls), or the OTN eConsult (defined as the times from the initial consult request to first contact and to when the consultation is provided). We will assess the time required for each consultation and follow-up encounter with the referring clinician. We will quantitatively measure satisfaction by measuring the proportion of GeriMedRisk recommendations that were executed by the referring clinician or the number of repeat referring clinicians. We define a priori threshold for acceptability as:

- The proportion of GeriMedRisk recommendations that were executed would be 60% or more [25, 26].
- The proportion of repeat referring clinicians per LTC site would be > 20% or more.

To identify human resources needs, we will measure the monthly number of consultations requiring physician support with geriatric medicine, clinical pharmacology, and geriatric psychiatry expertise. We will characterize the number of complex cases defined as a case either involving polypharmacy (four or more medications) [27] or requiring physician support or three or more follow-up encounters. We will also assess the financial feasibility of GeriMedRisk through salary and physician billings data [13].

#### Secondary outcomes

The core outcome set for effectiveness trials aimed at optimizing prescribing in older adults in care homes informed the selection of our secondary outcomes [28, 29]. These outcomes are clinically relevant, potentially modifiable, well-validated in the MDS-RAI 2.0, and in some cases, a surrogate marker of care [30–32]. For these same reasons, Health Quality Ontario, a provincial body that advises on the quality of health care provided, also uses these outcomes as long-term care quality indicators [32]. We will assess the completion of our secondary outcome data regarding each LTC site’s monthly rates of falls, hospital visits, and medication appropriateness. Using the Resident Assessment Index Minimum Data Set (RAI-MDS) 2.0, we will examine the following in the 1-year lookback period prior to the date of the intervention’s introduction and monthly thereafter [30]. We will examine each LTC site’s monthly incidence of falls and injurious falls and emergency hospital visits. We will include all-cause mortality and those associated with adverse drug events or falls [28]. We will characterize the proportion of LTC residents receiving potentially inappropriate medications defined by the Beers Criteria and STOPP/START criteria [4, 33], psychotropics (defined as a sedative-hypnotic, antidepressant, antianxiolytic, antipsychotic, lithium or antiseizure medication in the absence of a seizure diagnosis), and long-acting opioids.

### Randomization and allocation

Access to GeriMedRisk (intervention) will be sequentially and randomly rolled out over 8-week periods. The sequence will be randomly generated by a computer model and simple randomization techniques. The allocation sequence will be concealed in sequentially numbered, opaque, sealed envelopes that will be opened at the beginning of each step.

#### Blinding

The LTC sites (clusters) will be blinded to the allocation sequence. None of the LTC sites’ clinicians or administrators work across the sites. There will also be blinding of the data analysis. The secondary outcomes are systematically collected by nurses, nursing managers, and the LTC administration, in the RAI-MDS 2.0, as part of their mandated reporting to the Ministry of Health and Long-Term Care. By retrospectively collecting the secondary outcomes in the 12 months preceding the intervention (lookback period), this ensures blinded data collection of the LTC patient demographic and clinical variables for this period. During the study, the secondary outcome data will continue to be collected by the same LTC team. Since nurses and nursing managers involved in multidisciplinary patient care are key to geriatric patient care, it would not be ethical to blind them, or any clinicians, involved in the patient’s care. The LTC pharmacy records are administrative databases that automatically capture prescription and non-prescription medication use among LTC residents. Only aggregated, anonymized, and de-identified pharmacy records of the LTC will be used for the secondary outcomes. There will be no blinding procedure for patients and their caregivers. We will not remove blinding from individuals involved with data collection or analysis processes. For the qualitative evaluation, there will be no blinding procedure.

### Data collection

#### Primary and secondary outcomes

The primary outcomes of this study include the acceptability and feasibility of GeriMedRisk and the stepped-wedge cluster randomized controlled trial. These include factors influencing the successful implementation of the GeriMedRisk service in terms of feasibility and acceptability among users and non-users and staff providing the GeriMedRisk service. Secondary outcomes include the LTC site’s monthly rate of falls, hospital visits, and medication appropriateness. We will measure secondary outcomes in the 52-week period preceding the commencement of GeriMedRisk access (defined as day − 365 to the first day of access to GeriMedRisk) and after, on a monthly basis for each site. These pre-specified outcomes will be systematically collected from our project-specific database from the OTN eConsult database, telephone records, RAI-MDS 2.0, and physician billings.

The Ontario Telemedicine Network (OTN) database is an administrative database that contains physician contact information and specialty and dates of engagement with GeriMedRisk. We will utilize telephone records as measured by the Voice Over Internet Protocol telecommunications manager, Vertical Summit®, to capture call volumes, dropped calls, and ring, queue, and call times. We will use the RAI-MDS 2.0 data for each LTC site. This standardized assessment tool is routinely applied to all LTC residents in Ontario, Canada, upon LTC admission, readmission from the hospital, change in status, and on a quarterly basis [30–32, 34]. It captures demographical and clinical information that is frequently used in observational research and quality improvement initiatives. We will use de-identified and anonymized aggregate data from the RAI MDS for each LTC site. We will also use aggregated, anonymized, and de-identified LTC pharmacy records to obtain information about prescription and non-prescription medication use.

#### Qualitative outcomes

Qualitative data collection will be collected through semi-structured interviews, surveys, and staff consult reflection notes. A team of researchers, not involved in patient care, generated questionnaires based on the theoretical domains framework (TDF) for GeriMedRisk staff, LTC clinicians to understand the barriers, and facilitators to implementation and acceptability of GeriMedRisk [35]. The TDF-based questionnaire will be administered to GeriMedRisk staff throughout the intervention implementation. This survey will be sent to all LTC clinicians. We will choose a census sampling approach as the expected numbers of potential GeriMedRisk service users per LTC site will not be large. We will send the questionnaire to a convenience sample of non-referring clinicians, defined as clinicians who practice at the LTC but did not consult during the study period. A total of three email or telephone reminders for the survey will be sent out every 2 weeks to optimize the response rate. Additionally, GeriMedRisk staff will engage in and document self-reflection activities to inform challenges encountered and lessons learned.

### Data management

The OTN, GeriMedRisk clinical database, and pharmacy and electronic medical records are independently managed on secure servers. We will access these databases with encrypted password-protected computers stored in locked cabinets in a secure office. Qualitative study surveys to the referring clinicians, non-referring but eligible clinicians, and GeriMedRisk staff will be administered electronically, data will be de-identified, password-protected, and stored securely. No patient information will be included in the survey. Interviews will be recorded, and data analysis will be stored electronically as a password-protected document on a password-protected encrypted computer in a locked room.

### Statistical data analysis

#### Quantitative analysis

As the main objective of this study is to evaluate the feasibility of GeriMedRisk and its stepped-wedge cluster randomized trial design, we will describe the primary outcomes in a narrative analysis with 95% confidence intervals (CI) where appropriate. We define success or acceptability of GeriMedRisk as a composite of either 60% adherence to GeriMedRisk recommendations or at least 20% of each LTC sites’ physicians making more than one consult to GeriMedRisk during the study period (May 1, 2017, to December 31, 2017).

For the secondary outcomes, we will assess each LTC site’s outcomes, described as monthly rates, in the 12 months preceding the intervention (baseline) between clusters pre-intervention and calculate the intercluster variance. We will assess for intra- and intercluster differences in the secondary outcomes, described as each LTC site’s monthly rates, with weighted generalized linear mixed models. This definition will guide our interpretation of the study’s results and potential modifications to the GeriMedRisk intervention or subsequent larger efficacy trial’s protocol.

#### Sample size calculation

As this is a feasibility study, the primary outcomes include recruitment, acceptability, and time required for the intervention. We used the methods described by Hemming et al. to calculate the sample size for stepped-wedge cluster randomized controlled trials. While the current risk of adverse events at these sites is currently unknown, the emergency department visits rate for LTC in Ontario, Canada, is 25% [34]. Although LTC clinicians are the users of the intervention, patient outcomes remain of interest, particularly for the larger efficacy trial. In order to detect a decrease in ED visits by 20%, with four steps and one cluster introduced per step and a total sample size of 628 LTC patients, we estimate 181 patients per cluster for 80% power and 5% significance [36]. We estimate conservatively that we will need to have consults from 20% of each LTC’s clinicians (physicians, pharmacists, and nurse practitioners). The results of this study will inform our sample size calculation for the future larger efficacy trial.

#### Qualitative analysis

For our qualitative analysis, digital recording of the semi-structured interviews will be transformed into verbatim transcripts. Transcripts will be independently reviewed by two members of the research team and analyzed with the purpose of addressing the interview questions. Thematic analysis will be applied to qualitative data from all data collection methods (surveys, interviews, notes) to identify main ideas and themes that cross participant groups (physicians, nurses, pharmacists, GeriMedRisk staff). Quotes that are representative of typical statements given by participants will be extracted to support the themed interpretations. Descriptive data analyses (frequencies: counts, percents, means, SDs, minima, maxima) will be used to describe closed-ended survey data.

We will describe the challenges and accompanying solutions and decision-making process encountered during the study period. These will be informative during our study design process for the future efficacy trial protocol.

### Data monitoring and auditing

Although the GeriMedRisk intervention is in its early stages of development, we will still have a data monitoring committee consisting of JH, JT, SB, and DM. We will conduct an interim analysis after the enrollment of two sites (16 weeks). We will stop the trial if there is significant risk associated with the intervention. While this process will be independent of the trial sponsor (McMaster University), it will not be independent from the investigators due to feasibility.

### Harms

During our follow-up calls, we will actively collect, assess, report, and manage solicited and spontaneously reported adverse events and other unintended effects of the trial intervention or trial conduct. These include adverse drug events that occur as a result of GeriMedRisk’s suggestions.

### Knowledge dissemination

We plan to disseminate the results of this study in a high-impact peer-reviewed journal within 1 year after the trial end date. We will also share the results with our partners, public and patients, and relevant stakeholders. We will not use professional writers. All authors will fulfill authorship guidelines as defined by the International Committee of Medical Journal Editors. We consulted patients from the community and LTC about GeriMedRisk throughout its conception, study design, and materials (opt-out flyer).

## Discussion

If GeriMedRisk is a feasible intervention, it would be the first technology-based interdisciplinary geriatric pharmacology intervention in Canada. The risks of participating in this GeriMedRisk study include the clinician participant’s time involved for each consult and follow-up. Although each consult may take up to 45 min on the telephone, this time could be significantly decreased by contacting GeriMedRisk through the secure OTN eConsult platform. The benefits of participating in this study include access to geriatric pharmacology expertise in a timely fashion without patient travel to an urban academic health center. Participants will also have the opportunity to provide feedback about GeriMedRisk to help improve the service and its impact on real-world practice. Finally, participants will have access to a service that supports their continuing professional development through concise learning materials pertinent to their patients and quarterly summaries of their learning issues generated from their consults. There will be no cost to participants for these services. By understanding the experiences and perceptions of GeriMedRisk stakeholders, we will be able to provide generalizable information about feasibility and acceptability for the wider health community, refine both referral criteria and mode of delivery for the subsequent clinical trial, inform the potential rollout of the intervention to other healthcare sectors, and further develop the consultation service.

## Abbreviations

ADE: Adverse drug event
CI: Confidence interval
LTC: Long-term care
OTN: Ontario Telemedicine Network
RAI-MDS: Resident Assessment Index Minimum Data Set
